# *Galphimia glauca* and Natural Galphimines Block Schizophrenia-Like Symptoms Induced with Apomorphine and MK-801 in Mice

**DOI:** 10.1155/2019/8404258

**Published:** 2019-07-21

**Authors:** Mayra Alejandra Santillán-Urquiza, Maribel Herrera-Ruiz, Alejandro Zamilpa, Enrique Jiménez-Ferrer, Rubén Román-Ramos, Elian Yuritzi Alegría-Herrera, Jaime Tortoriello

**Affiliations:** ^1^Centro de Investigación Biomédica del Sur, Instituto Mexicano del Seguro Social (IMSS), Argentina 1, 62790 Xochitepec, Morelos, Mexico; ^2^Laboratorio de Farmacología, División de Ciencias Biológicas y de la Salud, Universidad Autónoma Metropolitana (UAM), Mexico City, Mexico

## Abstract

**Background:**

* Galphimia glauca* has been used for many years in Mexican Traditional Medicine to calm “insane people.” Triterpenes, known as galphimines, were identified in this species. One of them, Galphimine-B (G-B), acts selectively on dopaminergic neurons by antagonizing the effect of glutamate on NMDA receptors. The objective of this study was to evaluate the effect of* G. glauca* methanolic extract (GgMeOH), a Galphimine-Rich Fraction (GRF), as well as the galphimines G-A, G-B, and G-E, on the acute psychosis induced by Apomorphine (APO) in mice and on schizophrenia-like symptoms induced by subchronic administration of MK-801.

**Method:**

On the first day, ICR male mice were given GgMeOH, GRF, or one of the galphimines. On day two, animals were treated with APO, and on day 3, they were subjected to behavioral tests. In a second test, MK-801 was administered daily for 28 days. In this case, animals were treated daily with* G. glauca* products from day 9 to day 28 and then subjected to behavioral tests (passive avoidance test, open field test, forced swimming test, and social interaction test).

**Results:**

The increased number of stereotyped behaviors and grooming behaviors induced with APO were counteracted by all of the experimental treatments. MK-801 induced an increase in immobility time, which was blocked with G-B; GRF counteracted the decreased social interaction, and GgMeOH and GRF prevented the memory loss induced by MK-801.

**Conclusion:**

* G. glauca* and their derivatives products (GRF and galphimines) were able to interact with the dopaminergic and glutamatergic drugs and to block different behaviors associated with some of the positive, negative, and cognitive symptoms of induced schizophrenia in mice. It is necessary to continue with this research, in order to identify their mechanism of action.

## 1. Introduction

Schizophrenia is a chronic debilitating neurological disorder that is characterized by a complex and heterogeneous group of perceptual, cognitive, and emotional deficits [1], which affects approximately 1% of the world's population [2]. This disorder presents three domains of symptoms: positive symptoms (hallucination, delusions), negative symptoms (depression, social isolation, and apathy), and generalized cognitive deficits. Several antipsychotic drugs are used to treat schizophrenia, the majority of which act as dopamine D2 receptor antagonists, such as Haloperidol. Despite more than 50 years of research, some patients are resistant to first- and second-generation antipsychotics, and one-third of these patients show a poor response to antipsychotic treatment [3, 4]. These drugs are only partially effective and produce severe adverse effects on the motor system, behavior, and metabolism [5]. The current emphasis on pharmaceutical management of this condition is changing significantly. The search for new treatments aims to address the positive symptoms, cognitive problems, and negative symptoms associated with the disease, since these are the symptoms that most severely hinder patients' ability to carry out their normal activities and are most representative of the disability associated with this disorder [6]. These symptoms are not adequately treated with current antipsychotics or with any other therapy available [7]. It is therefore important to develop new, more effective therapeutic interventions, as well as valid and appropriate animal models, to identify novel therapies for schizophrenia [8].

Rodent models of schizophrenia are characterized mainly by behavioral changes such as hyperlocomotion, social deficiencies, and decreased working memory; these serve as indicators of behavioral anomalies and correspond to several domains of positive, negative, and cognitive deficiencies that occur in schizophrenic patients [9]. Apomorphine (APO) is able to induce symptoms similar to those of schizophrenia [10–12]. MK-801 and ketamine are antagonists of NMDA receptors and exhibit psychotomimetic properties and are therefore widely used as experimental inducers of schizophrenia symptoms in rodents [13, 14].

Aerial parts of the plant species* Galphimia glauca* have been widely used in Mexican Traditional Medicine for patients suffering from mental disorders, more specifically as a remedy for calming “insane people” [15]. From this plant, different nor-, seco-triterpenes have been isolated and were named galphimines. In a previous work, it was observed that the methanolic extract of* G. glauca* (GgMeOH), a Galphimine-Rich Fraction (GRF), and galphimines (G-A, G-B, and G-E) interact with the dopaminergic system, modifying the behavioral response. They strengthened the cataleptic effect produced by Haloperidol (HAL) and inhibited ketamine-induced depression and psychotic behavior in mice. In the case of GgMeOH and the GRF, they also blocked the cognitive deterioration induced by ketamine. It has been reported that the products obtained from* G. glauca* have a protective effect against some of the symptoms generated by ketamine and that GgMeOH and GRF are able to block the positive and cognitive symptoms of schizophrenia [13]. These findings are relevant because* G. glauca* acts by means of the dopaminergic and glutamatergic neurological pathways. The objective of this study was to evaluate the effect produced by the GgMeOH, GRF, as well as galphimines G-A, G-B, and G-E on the acute psychosis induced by APO (dopaminergic system), and schizophrenia-like symptoms induced with the subchronic administration of MK-801 (glutamatergic system), both in mice. The effects were evaluated by means of behavioral tests including the passive avoidance test for cognitive deterioration; open field test for positive symptoms [14, 16]; and the forced swimming test and social interaction test for negative symptoms.

## 2. Materials and Methods

### 2.1. Plant Material and Preparation of the Extract

The aerial parts of* Galphimia glauca* Cav. (Malpighiaceae) were identified by Abigail Aguilar-Contreras, M.Sc., Director of the IMSSM Herbarium. A voucher sample is found at the same Herbarium with the accession number IMSSM-11061. The dry plant material (10 kg) was ground in an electric mill to a particle size of <4-mm. Next, the material was extracted by maceration with n-hexane and then methanol for 2 days with three changes of solvent (at a plant:solvent ratio of 1:5). The solvent was eliminated in a rotary evaporator, and a dry methanolic extract of* G. glauca* (GgMeOH) was obtained with a yield of 18.5%. The extract was submitted to a chemical separation procedure to obtain the galphimines. Open-column chromatography was carried out with a silica-gel (Merck) normal phase and, as the mobile phase, a gradient system beginning with hexane: Ethyl Acetate (EtOAc) (7:3) with successively increasing EtOAc. Separation was monitored by thin-layer chromatography (TLC) to identify the fractions with the greatest amount of galphimines. The product obtained was submitted to High-Performance Liquid Chromatography (HPLC) until obtaining a Galphimine-Rich Fraction (GRF, 30 g) with a yield of 0.18%. From the GRF, we isolated each of the galphimines (G-A, G-B, and G-E). The galphimines were identified using HPLC. As reported previously [17], the GgMeOH and GRF were standardized in their content of G-A, G-B, and G-E.

### 2.2. Treatments Utilized in Animal Experiments

The compounds, extracts, and substances utilized included the following: GgMeOH (25 mg/kg, p.o.); GRF, G-A, G-B, and G-E (5 mg/kg, p.o.); Apomorphine hydrochloride (APO, 2.0 mg/kg, intraperitoneally, i.p.); haloperidol (HAL, 1.0 mg/kg, i.p.; 99%); vehicle (VEH, 1% Tween 20, p.o.); MK-801 hydrogen maleate (0.5 mg/kg, p.o; ≥98% (HPLC)); olanzapine (OLZ, positive control, 1 mg/kg, i.p.; Olanzapine ≥98% (HPLC)), and N-Methyl-D-Aspartate (NMDA, 1 mg/kg, p.o.; ≥98% (TLC), solid). All drugs were purchased from Sigma-Aldrich company. All treatments were administered at a volume of 100 *μ*L/10 g of animal weight.

### 2.3. Animals

We utilized male ICR mice weighing between 28 and 31 g kept under controlled conditions in a light/dark cycle (12/12 h), at a temperature of 20 ± 2°C, with free access to a special diet for rodents (Labdiet) and purified water. For the management and care of the laboratory animals, we followed Official Mexican Regulation NOM-062-ZOO-1999. The research was conducted in accordance with the internationally accepted principles for laboratory animal use and care as found in the National Institutes of Health (NIH) guidelines. Behavioral tests were performed in a soundproofed room with a video recording system; the experimenter avoided the use of perfumes or scented products and remained silent. The project was evaluated and authorized by the Ethics and Institutional Biosecurity Committee, registration number R-2014-1701-29.

### 2.4. Experimental Design

**Figure 1:**
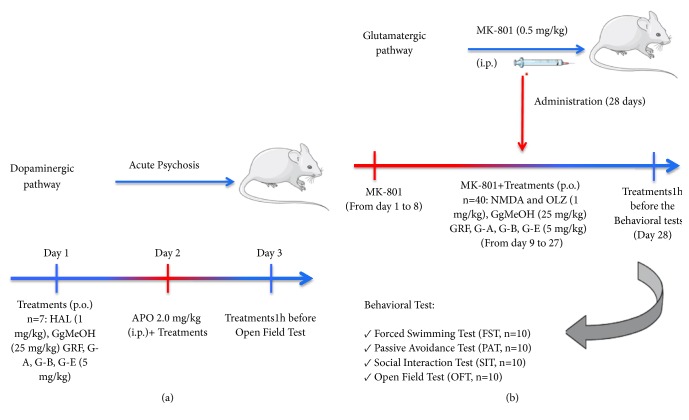
Diagram showing details of the two experiments evaluating the* Galphimia glauca* products,* G. glauca* methanolic extract, GgMeOH, the Galphimine-Rich Fraction, GRF, and of the different galphimines G-A, G-B, or G-E, on the dopaminergic (a) and glutamatergic (b) pathways.

In order to evaluate the effects of* G. glauca* products, acute schizophrenia-like behavior was induced by APO following the methods of Santillán-Urquiza, 2018 [17]. Seven mice were used for the open field test (OFT). The experimental procedure occurred over three days; on day 1, mice were given their corresponding experimental treatment; on day 2, all animals (except VEH group) were also given APO (2.0 mg/kg, i.p.). On day 3, animals were again given their experimental treatments, and 1 hour later, they were subjected to the behavioral test (Figure 1(a)).

In a second experiment, 40 different mice were used, 10 for each behavioral test (FST, SIT, OFT, passive avoidance test; PAT). With exception of the VEH group, all animals were given MK-801 (0.5 mg/kg, i.p.) daily for 27 days. From days 9 to 28, the animals were also treated with the corresponding experimental treatment (*G. glauca* methanolic extract, GgMeOH, the Galphimine-Rich Fraction, GRF, galphimines G-A, G-B, or G-E, N-Methyl-D-Aspartate, NMDA (the last was used as a control, because MK-801 works as NMDA receptor antagonist). On day 28, 1 h after administering the last treatment, animals were subjected to the corresponding behavioral test (FST, PAT, SIT, OFT, n=10) (Figure 1(b)).

#### 2.4.1. Forced Swimming Test (FST)

A protocol modified from Porsolt et al. was utilized [18]. Prior to the recorded test, mice underwent a training session that consisted of placing the mice individually for 15 min in a glass container with water at a depth of 15 cm, at a temperature of 25°  ± 2°C. This to ascertain whether the mouse identified that there was no way for it to escape. After administration of the treatments, the mice were placed in the glass container with water for 5 min. Total immobility time was measured for each mouse.

#### 2.4.2. Social Interaction Test (SIT)

The test equipment comprised a transparent acrylic box with three compartments with free access among them and has two small metal-wire cages for a single mouse located at the end of each compartment. The test begins with a habituation process, placing the experimental mouse in the central compartment, allowing him to explore for 10 min and to familiarize himself with the environment. After the 10 min habituation, a mouse is placed inside metal-wire cage and is called “familiar” (a mouse can be used from its same box), and again, the experimental mouse is allowed to explore for 5 min. On the day of the test, a “new mouse” that has never been exposed to the experimental mouse was placed in the remaining wire cage. The test is carried out over four sessions. In the first three sessions, the focal animal faces a familiar mouse and, during the course of these sessions the mouse adapts to that exposure (habituation). During the fourth exposure, the new mouse is placed inside the metal-wire cage (detoxification). In all of the sessions, the interaction time is measured, defined as the time that the focal animal spends approaching or sniffing the cage. Mice with a nonimpaired cognitive function will spend more time sniffing new mice than familiar ones, while those with a cognitive deficit will not distinguish between familiar and new mice (their time of “social interaction” will be similar) [19].

#### 2.4.3. Open Field Test (OFT)

The mice were placed on the OFT platform and their behavior was videotaped with a digital camera fixed above the field. Observation was maintained for 30 min, and we measured the following parameters: locomotion as Total crossings; Rearing; Stereotyped Behaviors (repetitive, involuntary movements with no obvious function, e.g. head-spinning); Grooming, and Time Spent on Grooming [20].

#### 2.4.4. Passive Avoidance Test (PAT)

This test consisted of placing the mouse in the lighted compartment, allowing it to explore for 1 min, and allowing it to move to the dark compartment (mice generally move spontaneously to the dark compartment, since direct light produces anxiety). The time that the mouse takes to move to the dark compartment (Entry Delay, ED) is measured; once the mouse is inside, the door is closed and the mouse receives an electric shock (0.4 mA, for 5 sec). After administration of the shock, the mouse was returned to its cage. Long-term memory is generally evaluated by repeating the test 24 h later. Thus, in the context of this experiment, we repeated the test one hour after administering the treatment. The mouse was placed into the lighted compartment and the door separating the two compartments was opened; then the mouse was observed for 480 sec to determine the time the mouse takes to enter the dark compartment (retention latency; RL), where shorter RL is an indicator of impaired memory [19].

### 2.5. Statistical Analysis

We used analysis of variance (ANOVA) followed by Dunnett post-hoc tests to determine whether there were differences among treatments in each behavioral test.* P*<0.05 was the significance threshold for all tests. In the results text and all figures and tables, *∗* indicates that the experimental treatment differed from the induced (APO or MK-801) group, and ^#^ indicates that the treatment differed from the VEH group. SPSS ver. 11.0 statistical software package was used for all analyses.

## 3. Results

### 3.1. Effects of GgMeOH, GRF, G-A, G-B, and G-E on APO-Induced Psychosis in Mice

#### 3.1.1. Behavior in the Open Field Test (OFT)

Results of the OFT are shown in Table 1. The administration of APO significantly modified the parameters associated with psychosis compared to VEH (*p* = 0.0001): the total crossings and rearings were decreased, stereotyped behaviors increased, number of grooming bouts decreased, and the time spent grooming increased. The administration of HAL decreased total crossings, rearings, stereotyped behaviors, and time spent on grooming with respect to the APO group (*p* = 0.0001), while number of grooming bouts was unchanged (*p* >0.05). The products derived from* G. glauca* had behavioral effects similar to HAL, with some exceptions. The administration of the different* G. glauca *products modified the effect of APO, significantly decreasing total crossings (*p* = 0.0001), stereotyped behaviors (*p* = 0.0001), and time spent on grooming (*p* = 0.0001). In the case of rearing, the treatments did not differ from APO (*p *>0.05). Finally, GgMeOH (25 mg/kg,* p* = 0.0001) increased grooming bouts, while G-B significantly decreased it (*p* = 0.0001) compared to the APO group.

### 3.2. Effect Produced by GgMeOH, GRF, G-A, G-B, and G-E on Mice with Schizophrenic-Like Symptoms Induced by MK-801

#### 3.2.1. Negative Symptoms Evaluated by Means of the Forced Swimming Test (FST)

**Figure 2:**
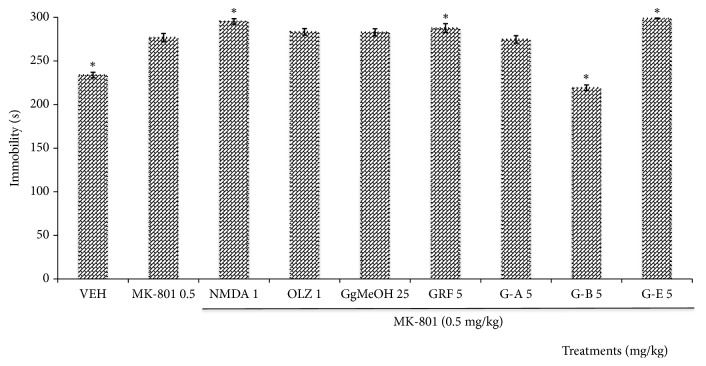
Effect of the administration of the methanolic extract of* Galphimia glauca* (GgMeOH, 25 mg/kg), of the Galphimine Rich Fraction (GRF), and of the different galphimines (G-A, G-B, and G-E) on mice with schizophrenic-like symptoms induced by MK-801 (0.5 mg/kg) in the Forced Swimming Test (FST). NMDA (1 mg/kg), OLZ = Olanzapine (1 mg/kg), and Vehicle (VEH) = negative control group. ANOVA, Dunnett post-hoc test, *∗* indicates significant difference compared to the MK-801 group at a significance threshold of* p*<0.05 (*n* = 10, results are means ± standard error [SEM]; DF = 9, F (54.14) 2.18, p = 0.0001).

Figure 2 illustrates that MK-801 induced an increase in the immobility time compared to healthy mice (VEH) when animals were exposed to FST (*p*= 0.001), while the coadministration of MK-801 and NMDA produced an even greater increase in this parameter with significant difference (*p* = 0.0001), when compared to the group with only MK-801. The administration of GRF (*p* = 0.015) and G-E (*p* = 0.0001) induced a similar behavior, and GgMeOH and OLZ did not differ significantly from the MK-801 group. Only G-B counteracted the effect produced by MK-801 (*p* = 0.0001).

#### 3.2.2. Negative Symptoms Associated with MK-801 on the Social Interaction Test (SIT)

**Figure 3:**
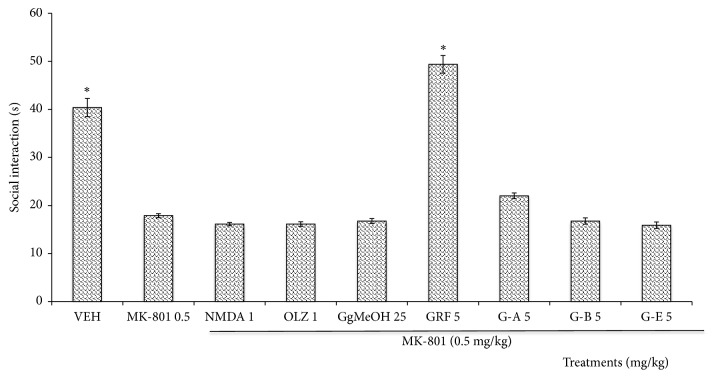
Effect of the administration of the methanolic extract from* Galphimia glauca* (GgMeOH, 25 mg/kg), the Galphimine-Rich Fraction (GRF), and the different galphimines (G-A, G-B, and G-E) on mice with schizophrenic-like symptoms induced by MK-801 (0.5 mg/kg) in the social interaction test (SIT). NMDA (1 mg/kg), OLZ = Olanzapine (1 mg/kg), and Vehicle (VEH) = negative control group. ANOVA, Dunnett post-hoc test. *∗* indicates significant difference compared to the MK-801 group at a significance threshold of* p*<0.05 (*n* = 10, results are means ± Standard Error [SEM]; FD = 9, F (72.19) 2.10, p = 0.0001).

In Figure 3, the administration of MK-801 reduced the time that mice spent sniffing the “new mouse” in comparison with VEH group (*p* = 0.0001). Among all of the treatments administered, only the GRF counteracted the effect of MK-801 significantly (*p* = 0.0001). None of the remaining treatments modified this parameter with respect to the MK-801 group (*p*>0.05).

#### 3.2.3. Cognitive Symptoms Associated with MK-801 in the Passive Avoidance Test (PAT)

**Figure 4:**
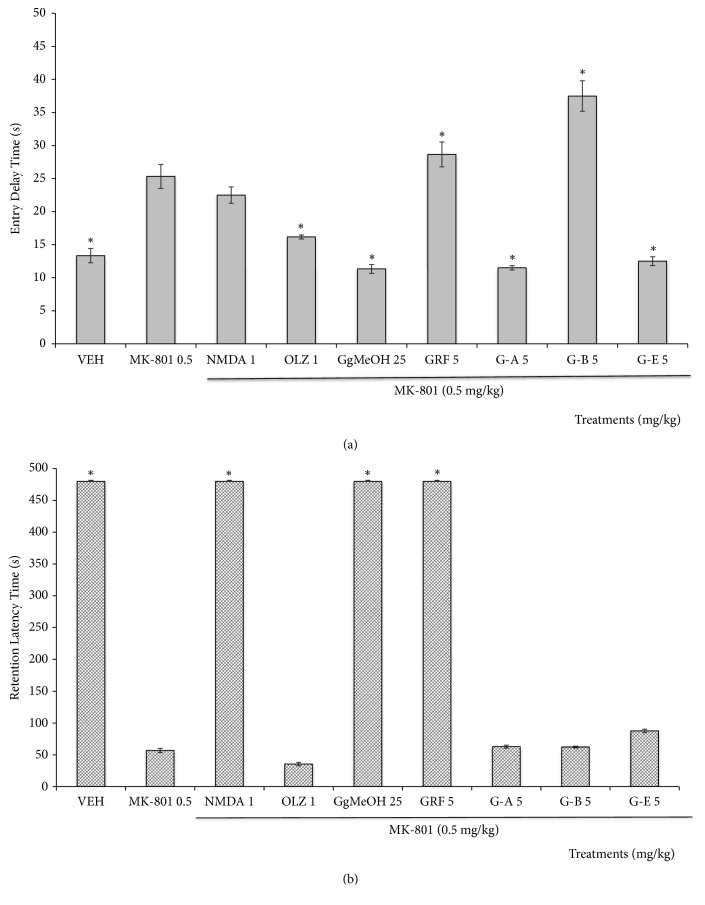
Effects of the administration of the methanolic extract of* Galphimia glauca* (GgMeOH, 25 mg/kg), the Galphimine-Rich Fraction (GRF), and the different galphimines (G-A, G-B, and G-E) on mice with schizophrenic-like symptoms induced by MK-801 (0.5 mg/kg) in Entry Delay Time (a) and retention latency time (b) in the passive avoidance test (PAT). NMDA (1 mg/kg), OLZ = Olanzapine (1 mg/kg), and Vehicle (VEH) = negative control group. ANOVA, Dunnett post-hoc test. *∗* indicates a significant difference from MK-801 group at a significance threshold of* p*<0.05 (*n* = 10, results are means ± Standard Error (SEM). Retention latency = (FD = 9, F (14888.38) 2.10, p = 0.0001), Entry Delay = (FD = 6, F (41.24) 2.10, p = 0.0001).

Figure 4(a) demonstrated that the training latency (Entry Delay, ED) of the VEH group (13.33 sec) was significantly lower (*p* = 0.0001) than that of the group treated with MK-801 (25.33 sec). OLZ, GgMeOH (*p* = 0.0001), G-A, and G-E (*p* = 0.0001) counteracted the effect produced by MK-801, while G-B induced a significant increase in ED compared to the MK-801 (*p* = 0.0001). On the other hand, NMDA (1.0 mg/kg) and GRF (5 mg/kg) did not show significant differences from the MK-801 group (*p* >0.05).

For the parameter RL (Figure 4(b)), the animals of the VEH group had an RL time of 480 sec. This value was significantly reduced by MK-801 (*p* = 0.0001, RL = 54.57 sec). The effect produced by MK-801 was significantly inhibited by NMDA (*p* = 0.0001), GgMeOH (*p* = 0.0001), and GRF (*p* = 0.0001), reaching values of RL similar to that of the VEH group. Animals treated with the atypical antipsychotic OLZ and with any of the galphimines (G-A, G-B, and G-E) did not differ from the MK-801 group (*p *>0.05).

#### 3.2.4. Positive Symptoms Associated with MK-801 in the Open Field Test (OFT)

Table 2 shows the results of the OFT. The administration of MK-801 induced a reduction in the number of total crossings and in the number of rearings and grooming bouts compared to the VEH group (*p* = 0.0001). They also spent a significantly longer time grooming with respect to the VEH group (*p* = 0.0001). Also, the mice treated with MK-801 presented stereotyped behaviors (an average of nine events), in contrast with those of the VEH group, which did not present this behavior (*p* = 0.0001). The NMDA significantly counteracted the number of stereotyped behaviors (*p* = 0.0001), but not any other behaviors. OLZ, on the other hand, significantly decreased all of the parameters evaluated in the OFT compared to mice that only received MK-801 (*p* = 0.0001).

All of the* G. glauca*-derived treatments except for GRF induced a decrease in total crossings and rearings compared to the VEH group (*p*= 0.0001), and none of the treatments demonstrated significant differences with the MK-801 group. On the other hand, time spent on grooming was decreased with the GgMeOH, GRF, and G-A treatments (*p* = 0.0001), while the other* G. glauca* products failed to induce significant differences in this parameter when compared with MK-801 (*p* >0.05). All treatments derived from* G. glauca *significantly reduced stereotyped behaviors compared to the MK-801 group (*p* = 0.0001).

## 4. Discussion

*Galphimia glauca* was able to modify in mice the behavioral manifestations induced by substances that produce symptoms similar to schizophrenia in animal models. Previously, it was demonstrated that GgMeOH, GRF, and galphimines G-A, G-B, and G-E modify the behavioral profile induced by ketamine in mice, and some of these products strengthen the activity of HAL, a typical antipsychotic that acts on dopaminergic receptors [17]. It has also been shown that G-B is capable of modifying the firing frequency of electrophysiological records of dopaminergic neurons in the VTA [21].

For evaluating treatments with possible antipsychotic activity, experiments evaluate behavioral parameters in rodents that resemble those that occur in humans. For example, locomotor hyperactivity, manifested by an increase in horizontal locomotor activity, rearing, or stereotyped behavior, is a parameter associated with the positive symptoms of schizophrenia and represent changes in dopaminergic neurotransmission, predominantly in the mesolimbic and nigrostriatal systems [22]. Thus, dopamine agonists can induce changes in these parameters in rodents.

Behavioral changes induced by selective or nonselective agonists of dopamine receptors, such as amphetamines and APO, and of substances acting on the glutamatergic neurotransmission system, such as MK-801, NMDA receptor antagonist, represent useful tools in the search for novel potential drugs [23, 24].

In this study, in order to expand on previously published results [23], the products obtained from* Galphimia glauca* were coadministered with APO (2.0 mg/kg), a synthetic derivative of morphine that has agonistic activity on the postsynaptic D1/D2 receptors of the nigrostriatal system. In rodents, APO induces a syndrome of stereotyped behavior as well as changes in motor function; information on this effect is plentiful and dates from the 1970s. The effects of APO are catalogued as part of the group of positive symptoms of schizophrenia, and it has been referenced that the diversity of behaviors caused by this substance depend strongly on the dose, administration scheme, route, and type of rodent [21, 25], explaining the wide diversity of responses observed in the scientific literature, especially in relation to locomotion [16].

In this study, APO decreased locomotion in the OFT, measured as the number of total crossings during 30 min. It is noteworthy that this was accompanied by a significant decrease in the number of rearings, the presence of stereotyped behavior (not observed in animals from the VEH group), a decrease in the number of grooming bouts, and an increase in time spent grooming. The literature notes that APO induces hyperkinesia, especially with doses in the range of 1.0 to 5.0 mg/kg; doses lower than 0.2 mg/kg have the opposite effect [24]. Several experiments showed that a dose of 0.1 mg/kg of APO in Sprague-Dawley rats reduced motor activity compared to rats receiving only saline solution [26]. In another test, carried out with Wistar rats, it is reported that the high dose of 2.0 mg/kg of APO causes hyperlocomotion, while lower doses do the opposite [27]. These results do not appear to agree with the effect that we obtained for APO at 2 mg/kg, in which this substance induced hypolocomotion of the mice during a 30-min observation period. It is noteworthy that the administration of APO occurred 24 h prior to the evaluation in the OFT. It must be considered that the behavioral results were a possible residual effect of this psychotropic drug, since pharmacokinetic studies indicate that i.v. administration of this substance (10 mg/kg) in ICR mice is rapidly distributed to the brain at a constant rate within 5 min [28]. It is expected, then, that, with the dose of 2 mg/kg i.p. administered to the mice, the animals undergo similar absorption kinetics and that, 24 h later, the APO has already been distributed and eliminated. Another possible explanation is based on the known habituation effect induced by some psychotropic substances such as APO. For example the administration of 2 mg/kg of this substance induces an increase in the total number of crosses in OFT, but only during first minutes of observation (0-5 min), while after 5-10 min, the number decreases and, in the period from 10 to 15 min, the number of crosses is significantly lower [29]. Another study revealed that i.p. administration of APO (2, 4, and 8 mg/kg) in C57BL6 mice resulted in an increase in the total number of crossings in the OFT, while in DBA/2 mice, a decrease of this parameter was observed [30]. Regarding rearing, in our work, APO produced an important and significant decrease of this event in the animals, while in another study of this drug (0.5 and 2.0 mg/kg) in rats 2 days after administration the rearing number was lower [31]. The response of the animals that received some of the products from* G. glauca *or with HAL was that of diminishing the total number of crossings in the OFT induced with APO, while rearing was not modified by any of the* G. glauca* products.

As already mentioned, APO induces in rats a “stereotyped behavior syndrome,” characterized by repetitive movements of the head and front legs and by manifestations of sniffing, licking, biting, etc. [32]. The stereotyped behavior induced by APO in mice was significantly reduced with GgMeOH, GRF, G-A, G-B, and G-E. This effect had been observed previously, but with stereotyped behaviors induced by a glutamatergic drug such as ketamine [17]. That is to say,* Galphimia glauca* counteracts the psychotic behavior induced by dopaminergic as well as by glutamatergic mediators.

The grooming behavior in mice treated with APO decreased significantly compared with that of the mice without APO, but the total time spent grooming was 362 sec, such that each grooming event lasted, on average, about 120 sec; mice treated with APO and then GRF, G-A, G-B, or G-E did not modify the number of grooming events, although they reduced the time during which they were performed. The only product that slightly counteracted the effect of the dopaminergic drug was GgMeOH, which increased grooming by about 8 sec. It has been reported that the stimulation of the D1 receptors increases grooming in rodents, while the stimulation of the D2 receptors decreases it. This is consistent with the effect observed with APO, in that this substance possesses a preference for D2 and exerts an effect that is inversely proportional to the dose in terms of the number of grooming bouts [33].

The interaction of each of the products derived from* G. glauca* with MK-801 was also evaluated; this noncompetitive antagonist of NMDA has become a useful tool in exploring the participation of the glutamatergic system in schizophrenia [34]. Administration of MK-801 (0.5 mg/kg) for 28 days induced hypolocomotion in mice, which was measured for 30 min in the OFT, since the parameters of total crossings and rearings were lower than those of healthy animals. This behavior coincides with that reported by other authors. For example, Li et al. described that the administration of 0.25 mg/kg, also for 28 days in rats, induced a decrease in the distance traveled in an open field, implying a decrease in motor activity [35]. Together with this behavior, we also found an increase in the number of stereotyped behaviors, a decrease in the number of grooming bouts, but an increase in the time that the mice spent grooming. It has been proposed that grooming is part of the battery of evidence of anxious behavior in animals [36], a symptom that also occurs in patients with psychosis. In the experiment of Li et al. (2013), the authors indicated that MK-801 (a noncompetitive NMDA antagonist with high affinity for rNMDA) causes a decrease in the time that mice spend in the center of the open field, more evidence of increased anxiety-like behavior. Regarding stereotyped behavior, administration of MK-801 (0.6 mg/kg) for 7 days in Swiss mice gave rise to a significant increase in the number of stereotyped behaviors, with a maximal-response peak at 30 min of observation [37]. The hypothesis, which involves the glutamatergic system as a participant in the pathophysiology of schizophrenia, is based on the hypofunction of the NMDA receptor. Thus, glutamatergic antagonists such as ketamine and MK-801 are widely employed as pharmacological tools for inducing endophenotypes or behaviors that are associated with schizophrenia, such as motor changes, stereotyped behavior, and cognitive decline [38]. In this respect, NMDA administered orally did not change the motor behavior of mice compared with that of those receiving only MK-801, although it did decrease stereotyped behavior and increase the time spent grooming, while OLZ increases the hypolocomotor effect by decreasing the total crossings and rearing in OFT and decreases stereotyped behavior, grooming, and time spend on grooming. OLZ is an atypical antipsychotic that exhibits an affinity for different neurotransmitters receptors, including serotonin, norepinephrine, and dopamine [39]. Similar to our results, Clozapine (another atypical antipsychotic) further reduced the motor activity and exploratory behavior (rearing) of neonatal mice that had received MK-801 (0.25 mg/kg) days 7-10 after birth [40].

Regarding the response with NMDA, this would be expected to block the effects induced by MK-801 in all of the observed psychotic endotypes in the open field; however, the sole change was a decrease of stereotyped behavior and time spent grooming. It is argued that the modification of motor activity caused by NMDA receptor dysfunction is associated with changes in the signaling of neurotransmitters, such as dopamine, serotonin, acetylcholine, and norepinephrine [40, 41]. In this way, a complex interaction is established, in which the NMDA (1 mg/kg) was not able to modulate and therefore did not counteract, in our experimental design, all of the events caused by its antagonist, at least in terms of motor functions.

In addition to positive symptoms, patients with schizophrenia exhibit negative symptoms, which are manifested as emotional expressions, deficits in social interactions, and depression, among others. Some behavioral tests attempt to emulate those symptoms in rodents. For example, Pérez et al. utilized Phencyclidine (PCP) as a pharmacological tool, basing their experiment on the fact that the prolonged consumption of PCP in humans produces negative symptoms, including depression [41]. Subsequently, under the same principle, Langen et al. proposed to evaluate depression in the FST by administering MK-801 for 15 days (0.2 and 0.3 mg/kg) instead of PCP. The authors showed that MK-801 was able to increase immobility time in the FST, a parameter that was evaluated 3 days after the last administration of this NMDA antagonist, while locomotion was not affected when measured in the OFT after 4 days [42]. Here, we found similar results when the mice were given MK-801 daily (0.5 mg/kg, for 28 days). The animals demonstrated an increase in immobility time in the FST test, but, unlike in the Langen trial (2012), in our work there was a decrease in locomotion during the OFT. Despite these differences, it is clear that the continuous blockade on NMDA receptors induces behaviors in mice that are similar to the negative and cognitive symptoms of psychosis.

It has been observed that* G. glauca* and its products, including the isolated compounds, counteract the effect of ketamine (50 mg/kg) administered 24 h before the behavioral test on the parameter of immobility [17], while, in the present work, the prolonged administration of GRF and G-E (5.0 mg/kg) in the test with MK-801 produced an effect similar to that of NMDA. This led to a greater increase in immobility, while G-B at the same dose induced a decrease in the same parameter. This means that G-B was the only product that counteracted the effect of MK-801, neither OLZ, GgMeOH, nor G-A changed the behavior.

It is evident that the 28-day regimen of the administration of MK-801 induces a decrease in the socialization behavior of the mice (evaluated by the SIT) and in memory decline (evaluated by means of the PAT). However, in terms of* G. glauca*, the sole product that modified the interaction behavior of mice was GRF. Also, GRF together with the GgMeOH extract counteracted the memory decline induced with MK-801. With other* G. glauca* products, although they increased retention latency with respect to the MK-801 group, this behavior does not appear to indicate an effect of improvement of memory. The products derived from* G. glauca* induced different effects, but it is noteworthy that all of these increased total crossings, causing the animals to behave, in this parameter, as the VEH group did; stereotyped behavior also decreased. While the number of grooming bouts does not change with these treatments, GgMeOH, GRF, and G-A did reduce the time that the animals invested in grooming.

## 5. Conclusion

These results show that the endotypes associated with psychosis that were emulated with dopaminergic and glutamatergic drugs were largely counteracted by the products derived from* G. glauca* either acutely with ketamine (demonstrated previously) and APO or chronically with MK-801. It is therefore possible to conclude that the products obtained from* G. glauca* are able to interact with the dopaminergic and glutamatergic systems by blocking the positive, negative, and cognitive induced-symptoms of schizophrenia.

## Figures and Tables

**Table 1:** Effect of the administration of the methanolic extract of *Galphimia glauca* (GgMeOH), the Galphimine-Rich Fraction (GRF), and the different galphimines (G-A, G-B, and G-E) on the acute symptoms of Apomorphine (APO)-induced schizophrenia in mice during the Open Field Test (OFT) (observation time: 30 min).

Treatments (mg/kg)	Total Crossings	Rearing	Stereotyped Behaviors	Grooming	Time Spent on Grooming (sec)
VEH	446 ± 13.6*∗*	247 ± 9.9*∗*	0 ± 0.0*∗*	22 ± 0.3*∗*	241 ± 3.8*∗*

APO 2	380 ± 16.9	48 ± 7.5	7 ± 0.2	3 ± 0.2	362 ± 7.0
HAL 1	109 ± 11.2*∗*	4 ± 0.4*∗*	0 ± 0.0*∗*	3 ± 0.3	27 ± 1.5*∗*
GgMeOH 25	222 ± 11.3*∗*	41 ± 5.0	2 ± 0.2*∗*	8 ± 0.6*∗*	69 ± 7.9*∗*
GRF 5	284 ± 12.4*∗*	47 ± 6.3	2 ± 0.2*∗*	4 ± 0.2	30 ± 3.1*∗*
G-A 5	304 ± 9.1*∗*	48 ± 6.2	2 ± 0.0*∗*	4 ± 0.5	36 ± 3.8*∗*
G-B 5	236 ± 12.5*∗*	54 ± 6.6	1 ± 0.2*∗*	1 ± 0.0*∗*	11 ± 1.2*∗*
G-E 5	200 ± 16.5*∗*	66 ± 3.6	2 ± 0.4*∗*	2 ± 0.0	12 ± 1.4*∗*

Total crossings, rearings, stereotyped behaviors and grooming (number of events), time spent on grooming (seconds). Apomorphine (APO); Vehicle (VEH): mice with 1% Tween 20; HAL: Haloperidol; GgMeOH: *Galphimia glauca* methanolic extract; GRF: Galphimine-Rich Fraction; G-A, G-B, and G-E (galphimines) from *Galphimia glauca*. ANOVA, Dunnett post-hoc test (results are means ± Standard Error (SEM), *∗*p ≤0.05 was considered significant difference in comparison with APO. Total crossings = (FD = 6, F (46.61) 2.25, p = 0.0001), rearings= (FD = 6, F (114.70) 2.25, p = 0.0001), grooming = (FD = 6, F (239.8) 2.25, p = 0.0001), time spent on grooming = (FD = 6, F (627.9) 2.25, p = 0.0001).

**Table 2:** Effects of the oral administration of the methanolic extract of *Galphimia glauca* (GgMeOH, 25 mg/kg), the Galphimine-Rich Fraction (GRF), and the different galphimines (G-A, G-B, and G-E) on mice with schizophrenic-like symptoms induced by the chronic administration of MK-801 evaluated by means of the Open field test (OFT) for 30 min.

Treatments (mg/kg)	Total Crossings	Rearing	Stereotyped Behaviors	Grooming	Time Spent on Grooming (sec)
VEH	385 ± 11.1*∗*	252 ± 8.0*∗*	0 ± 0.0*∗*	24 ± 0.8*∗*	236 ± 6.9*∗*

MK-801 (0.5)	183 ± 7.2	72 ± 5.2	9 ± 0.8	16 ± 1.8	424 ± 11.5
NMDA (1.0)	180 ± 11.0	78 ± 9.3	3 ± 0.6*∗*	18 ± 1.3	549 ± 15.1*∗*
OLZ (1.0)	100 ± 6.8*∗*	25 ± 3.5*∗*	1 ± 0.2*∗*	7 ± 0.7*∗*	160 ± 6.8*∗*
GgMeOH (25.0)	241 ± 13.5*∗*	71 ± 5.5	4 ± 0.3*∗*	13 ± 0.5	371 ± 12.2*∗*
GRF (5.0)	223 ± 10.5	89 ± 6.6	3 ± 0.3*∗*	17 ± 0.7	103 ± 10.8*∗*
G-A (5.0)	331 ± 11.3*∗*	99 ± 8.0	4 ± 0.4*∗*	16 ± 2.0	295 ± 11.1*∗*
G-B (5.0)	232 ± 13.2*∗*	98 ± 8.0	3 ± 0.3*∗*	13 ± 1.2	414 ± 9.7
G-E (5.0)	229 ± 11.1*∗*	93 ± 9.0	2 ± 0.2*∗*	20 ± 1.1	392 ± 15.3

Total crossings, rearings, stereotyped behaviors and grooming bouts, time spent grooming (seconds). MK-801; Vehicle (VEH) = mice with Tween 20 1%; OLZ = Olanzapine; GgMeOH = methanolic extract from *Galphimia glauca*; GRF = Galphimine-Rich Fraction. ANOVA, Dunnett post-hoc test. Results are means ± Standard Error (SEM). *∗* indicates a significant difference from MK-801 at a significance threshold of *p*<0.05. total crossings = (FD = 9, F (77.65) 2.10, p = 0.0001), rearing = (FD = 9, F (67.26) 2.10, p = 0.0001), grooming = (FD = 9, F (14.23) 2.10, p = 0.0001), time spent on grooming = (FD = 9, F (627.9) 2.25, p = 0.0001).

## Data Availability

Additional data to the manuscript will be available by request.

## Abbreviations

APO: Apomorphine
ED: Entry delay
EtOAc: Ethyl Acetate
FST: Forced swimming test
G-A: Galphimine-A
G-B: Galphimine-B
G-E: Galphimine-E
GgMeOH: * Galphimia glauca* methanolic extract
GRF: Galphimine rich fraction
HAL: Haloperidol
HPLC: High-performance liquid chromatography
NMDA: N-Methyl-D-Aspartate
OFT: Open field test
OLZ: Olanzapine
PAT: Passive avoidance test
RL: Retention latency
SIT: Social interaction test
TLC: Thin-layer chromatography
VEH: Vehicle.
